# Switched photocurrent direction in Au/TiO_2_ bilayer thin films

**DOI:** 10.1038/srep10852

**Published:** 2015-06-01

**Authors:** Hongjun Chen, Gang Liu, Lianzhou Wang

**Affiliations:** 1 Nanomaterials Centre, School of Chemical Engineering and Australian Institute for Bioengineering and Nanotechnology, The University of Queensland, QLD 4072, Australia; 2 Shenyang National Laboratory for Materials Science, Institute of Metal Research, Chinese Academy of Sciences, 72 Wenhua Road, Shenyang 110016, China

## Abstract

Switched photocurrent direction in photoelectrodes is a very interesting phenomenon and has demonstrated their potentials in important applications including photodiodes, phototransistors, light-driven sensors and biosensors. However, the design and mechanism understanding of such photoelectrodes remain challenging to date. Here we report a new phenomenon of sequence-driven the photocurrent direction on a simple bilayer structure of 5 nm thick Au and 10 nm TiO_2_ under visible-light irradiation. It is found that when Au layer are deposited as the outer layer on TiO_2_ coated fluorine doped tin oxide (FTO) substrate (designated as FTO/TiO_2_/Au), anodic photocurrent is obtained due to the band bending formed at the electrode-electrolyte interface. Interestingly, simply swapping the deposition sequence of Au and TiO_2_ leads to cathodic photocurrent on FTO/Au/TiO_2_ electrode. Characterization and calculations on the photoelectrode reveals that the photogenerated electrons can be easily trapped in the energy well formed between the band bending and the Schottky contact, which allows electronic tunnelling through the 1.6 nm thick space charge layer, resulting in a unique anodic to cathodic photocurrent conversion. The understanding of this new phenomenon can be important for designing new generation optoelectronic converting devices in a low-cost and facile manner.

TiO_2_ has been extensively investigated for various potential applications including photocatalytic water splitting, environmental remediation, and third generation solar cells since the discovery of its photoelectrochemical water splitting phenomenon^1^. As an n-type semiconductor with a wide band gap around 3 ~ 3.2 eV, TiO_2_ only absorbs UV light, which significantly limits its capability in solar energy utilization. A variety of strategies have been explored to extend the absorption of TiO_2_ to visible regime and to improve its visible light photo-activity. Recently, a new approach involving plasmonic metal nanoparticles (NPs) like Au and Ag to enhance the visible-light photoactivity of wide bandgap semiconductors has attracted much attention^2^,^3^. These plasmonic metal NPs support collective electron oscillations, known as surface plasmon resonance (SPR). The intrinsic property of SPR makes the plasmonic metal NPs act as an antenna to receive the optical energy, which remarkably improves the visible-light photocatalytic properties of the host semiconductors^2^. The function of the plasmonic metal NPs in the photoexcited process is similar to the role of a photosensitizer, but more photostable and environmentally friendly than commonly used quantum dots^4^. Some research works have reported the plasmonic-enhanced effect of Au NPs on TiO_2_^5^,^6^,^7^,^8^, but the underlying mechanisms are still under debate. The main reason lies in that most of Au/TiO_2_ systems^7^,^8^ employed simply mixed Au and TiO_2_ NPs and there is no clear continuous interface between the two components, which apparently increase the difficulty for elucidating the mechanism.

The control of photocurrent direction on photoelectrodes is an intriguing phenomenon, which has been reported on some molecules, polymers and hybrid photocatalysts systems^9^,^10^,^11^,^12^,^13^,^14^,^15^,^16^,^17^,^18^,^19^. In order to tune the photocurrent direction, external factors including potential, wavelength, and pH value are normally applied in these systems to trigger the conversion. For example, graphene doped graphitic carbon nitride exhibited an ambipolar behavior under certain bias voltages due to the different concentration of oxygen defects in the doped graphene^13^. We recently reported an n-type to p-type switchable photoelectrode assembled from exfoliated TiO_2_ nanosheets and polyaniline, in which the structure of polyaniline under different pH values plays a dominant role in determining the photocurrent direction^14^. These systems have demonstrated their potentials in important optoelectronic fields like photodiodes, phototransistors, light-driven sensors and biosensors^9^,^10^,^11^,^12^,^13^,^14^,^15^,^16^,^17^,^18^,^19^. Furthermore, if two or more stimuli are available in one system, these variables can also be combined for the design of programmable chemical logic gates^18^ with the potential applications in the fields of artificial intelligence, information technology, and computer science. In light of a variety of potential applications, it is important to develop new switchable photocurrent systems utilizing stable inorganic materials.

In this study, we design a very simple bilayer thin film structure using an electron-beam (E-beam) technique. The FTO glasses were coated with 5 nm thick Au and 10 nm TiO_2_ in a swapped sequence as photoelectrodes, which not only exhibit visible-light photoactivity, but also demonstrate a unique *anodic vs. cathodic* switched photocurrent under visible-light irradiation, simply depending on the film deposition sequence. The electronic tunnelling through the band bending was proposed to explain this novel phenomenon. The new finding based on such a simple bilayer structure design may shed light on the development of new types of photo-induced devices and optical logic gating devices by simply controlling the deposition sequence.

## Results

### Characterization of Au/TiO_2_ bilayer structures

**Figure 1:**
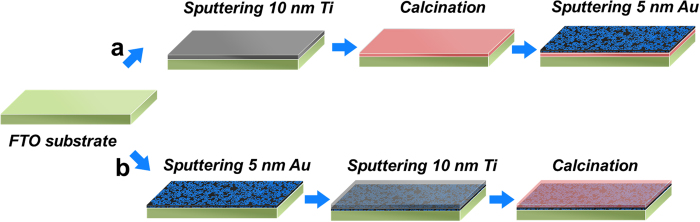
**The fabrication procedure of Au/TiO**_**2**_ **bilayer structures on FTO substrates.** (**a**) FTO/TiO_2_/Au and (**b**) FTO/Au/TiO_2_ photoelectrodes.

The hybrid photoelectrodes were prepared on FTO substrates by an E-beam sputtering technique. As shown in Fig. 1a, in a typical process for FTO/TiO_2_/Au photoelectrode, the FTO substrate was firstly sputtered with 10 nm of Ti, then calcined at 450 °C for 1 hour in air to transform metallic Ti into TiO_2_, and the last step was the continual sputtering of 5 nm of an Au top layer. The hybrid FTO/Au/TiO_2_ photoelectrode was fabricated simply using the reverse sequence as that of FTO/TiO_2_/Au photoelectrode, as shown in Fig. 1b.

**Figure 2:**
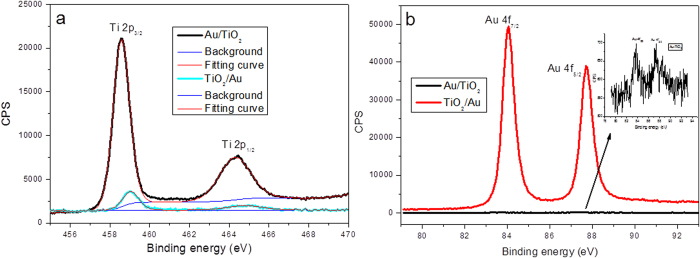
High resolution XPS spectra of (**a**) Ti and (**b**) Au for FTO/Au/TiO_2_ (black) and FTO/TiO_2_/Au (red) photoelectrodes. The inset in Fig. 2b is the enlarged XPS spectra of Au for FTO/Au/TiO_2_ photoelectrode.

X-ray photoelectron spectroscopy (XPS) analysis was used to confirm the bilayer structure and chemical states of the resultant photoelectrodes. The Ti 2p3/2 peaks centred at binding energies of 458.6 and 458.9 eV were revealed for both FTO/TiO_2_/Au and FTO/Au/TiO_2_ photoelectrodes (Fig. 2a). After fitting, it is found that the Ti 2p_1/2_ and Ti 2p3/2 spectra are nearly symmetric. Further investigation on the the binding energy and the difference of binding energy between Ti 2p1/2 and Ti 2p3/2 (around 6 eV), it is found that they are in good agreement with the typical values for TiO_2_^20^, indicating the full oxidation of metallic Ti into TiO_2_ upon calcination. As shown in Fig. 2b, the high-resolution XPS spectra of Au indicate that the binding energy of Au 4f5/2 and Au 4f7/2 doublet with binding energies of 87.7 and 84 eV respectively for FTO/TiO_2_/Au, which are typical value for Au in zero state^21^,^22^. In comparison, the XPS intensity of Au component in FTO/Au/TiO_2_ photoelectrode is too weak and the binding energy of Au 4f5/2 and Au 4f7/2 locates at 87.4 and 83.7 eV, respectively. The binding energy of Au in FTO/Au/TiO_2_ is 0.3 eV lower than that of FTO/TiO_2_/Au, suggesting the interaction between Au and TiO_2_ makes the binding energy of Au in Au/TiO_2_ shift a little bit negative. The intensity difference of Ti and Au elements in two photoelectrodes clearly reflects the top layers in the sequence-swapped bilayer thin films, i.e. one being Au, and the other TiO_2_. It is noted that the Au layer will not form continuous film due to its ultrathin feature. To further verify the bilayer structure in the films, XPS depth profiles were also conducted (Figure S1). The changes of at% Ti and at% Au in the course of Ar etching process in two types of films verified that the Au and TiO_2_ bilayer films were prepared on the FTO substrates with an opposite sequence. Note that the atomic ratio between O and Ti is more than 2 and the extra O content probably comes from the surface absorbed O_2_ or CO_2_ and the FTO substrates. Other characterizations including UV-Vis (Figure S2), XRD (Figure S3) and SEM (Figure S4) results also confirm the bilayer structures in the two photoelectrodes (Supporting information).

### Photoelectrochemical behavior of the Au/TiO_2_ bilayer photoelectrodes

**Figure 3:**
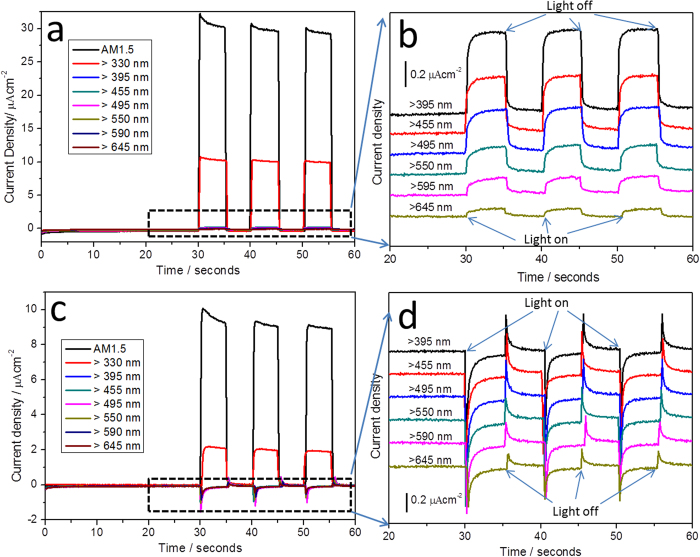
I-t curves of FTO/TiO_2_/Au (**a**) and FTO/Au/TiO_2_ (**c**) photoelectrodes under chopped light with different filter wavelengths. (**b**) and (**d**) are the enlarged I-t curves for the dashed rectangular part in (**a**) and (**c**). Applied potential: −0.1 V vs. Ag/AgCl.

The photoactivity of both photoelectrodes was evaluated using an amperometric current-time (I-t) technique. The measurements were conducted with three-electrode configuration at −0.1 V vs. Ag/AgCl in 1 M NaOH electrolyte. As depicted in Fig. 3a, the FTO/TiO_2_/Au photoelectrode exhibits anodic photocurrent under applied chopped light of all wavelengths. As the filter wavelength increased from >330 to >645 nm, the anodic photocurrent density of FTO/TiO_2_/Au photoelectrode gradually reduced (Fig. 3b). Similar to the FTO/TiO_2_/Au photoelectrode, the FTO/Au/TiO_2_ photoelectrode also exhibited anodic photo-current under AM 1.5 and >330 nm illumination, as shown in Fig. 3c. When the irradiated wavelength increased from >395 to >645 nm, interestingly, cathodic photocurrent with huge spike transient photocurrent were observed, totally different from that of FTO/TiO_2_/Au photoelectrode. More interestingly, the photocurrent density has no obvious change when the filter wavelength increased from >395 to >645 nm. In order to elucidate this unique cathodic photocurrent, two control experiments were carried out under the same experimental condition. As shown in Figure S5, the anodic photocurrent of 10 nm of TiO_2_ almost disappeared when the filter wavelength was larger than 495 nm, which is in agreement with the behaviour of UV-active only TiO_2_. The other control experiment was conducted using 5 nm of Au NPs on FTO as the photoelectrode shown in Figure S6a. The FTO/Au has almost no response under different filter wavelength irradiation except under AM 1.5. The weak photore-sponse under AM 1.5 mainly comes from the underlying FTO substrate (Figure S6b). From the comparison, it is very clear that the photocurrent under visible-light irradiation should be a synergetic effect of Au NPs and TiO_2_. The anodic photocurrents of both hybrid photoelectrodes mainly come from the TiO_2_ under AM 1.5 or when the irradiation wavelength >330 nm. Meanwhile, the photocurrent is almost zero during the first 30 seconds in Fig. 3a,c, which can rule out the possibility of the leaking photocurrent from the environment. Here, the number of the generated electrons can be roughly estimated based on the equation:
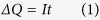
Where *ΔQ, I,* and *t* are the amount of charge density passing, current density, and time respectively. If taking the FTO/TiO_2_/Au photoelectrode under >455 nm illumination as a representative case (red curve in Fig. 3b), the stationary photocurrent density generated is around 0.45 μA cm^−2^. So the charge density calculated is around 0.45 μC cm^−2^ per second. Based on the charge of one electron is q = −1.6 × 10^−19^ C, the number of electrons generated is around 2.81 × 10^12^ per square centimetre per second. Under the same experimental condition, the stationary photocurrent density of FTO/Au/TiO_2_ photoelectrode can only produce 0.041 μA cm^−2^, therefore, 2.56 × 10^11^ of electrons generate per square centimetres per second, which is 10 times less than that of FTO/TiO_2_/Au photoelectrode.

When the photoelectrodes are excited by UV light, the photon-generated electrons are injected into the conduction band of TiO_2_, and leave the holes at valence band. Due to the band bending, the electrons transfer to the counter electrode through the outer circuit, while the holes are directly involved in the oxidation reaction at the interface of photoelectrode and solution. Thus, anodic photocurrent is produced on both photoelectrodes. The comparable photocurrent density between benchmark FTO/TiO_2_ and hybrid photoelectrodes especially FTO/TiO_2_/Au photoelectrode under UV light can be the evidence for explaining the above-mentioned charge-transfer process. On the other hand, both photoelectrodes exhibited visible-light photoactivity due to the plasmonic enhanced effect of Au NPs, which is also reflected in the UV-visible absorption of both photoelectrodes (see Figure S2). However, the enhancement is more noticeable for FTO/TiO_2_/Au than FTO/Au/TiO_2_ photoelectrode. It seems that the plasmonic enhanced effect of Au NPs is not taking effect under UV illumination; a similar phenomenon was also reported recently^6^.

**Figure 4:**
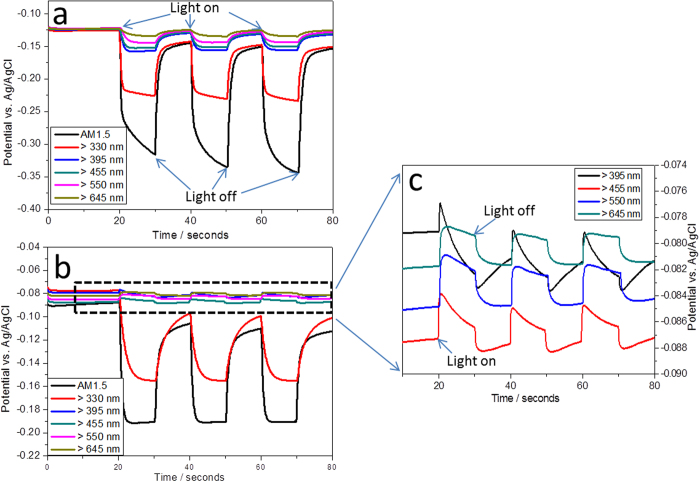
The open circuit potentials (OCPs) of photoelectrodes. (**a**) FTO/TiO_2_/Au and (**b**) FTO/Au/TiO_2_ photoelectrodes under chopped light with different filter wavelengths. (**c**) is the enlarged figure for the dashed rectangular part in (**b**).

The open circuit potential (OCP) is the potential difference between the working and reference electrodes when the current in the outer circuit is zero. Therefore, if the photoelectrode is irradiated with light, the change of the OCP should be the direct reflection of the generated photovoltage. The OCPs of both photoelectrodes were also measured under chopped light with different filter wavelengths. As shown in Fig. 4a, when changing the filter wavelength from AM1.5 to >645 nm, all the generated photovoltages of FTO/TiO_2_/Au photoelectrode are negative and gradually decrease, indicating the FTO/TiO_2_/Au photoelectrodes are accumulating negative charge. As presented in Fig. 4b,c, when an FTO/Au/TiO_2_ photoelectrode is illuminated under AM 1.5 and >330 nm, the photovoltages generated are also negative, which is similar to that of FTO/TiO_2_/Au photoelectrode. When the filter wavelength increases to >395 nm illumination, the generated photovoltage is initially positive and then gradually decreases to more negative values. As the filter wavelength continually increases from >455 to >645 nm, the photovoltages of Au/TiO_2_ photoelectrode are totally changed into positive values, suggesting positive charges are accumulated. The photovoltages obtained under different wavelengths of illumination are directly related to the photocurrents measured in Fig. 3. When the visible-light photocurrent changes from anodic to cathodic for FTO/TiO_2_/Au and FTO/Au/TiO_2_, respectively, the value of photovoltage also correspondingly changes from negative to positive. This phenomenon indicates the transferring direction of photo-generated electrons reversed.

## Discussion

### Mott-Schottky curve and visible-light excited charge-transferred process

**Figure 5:**
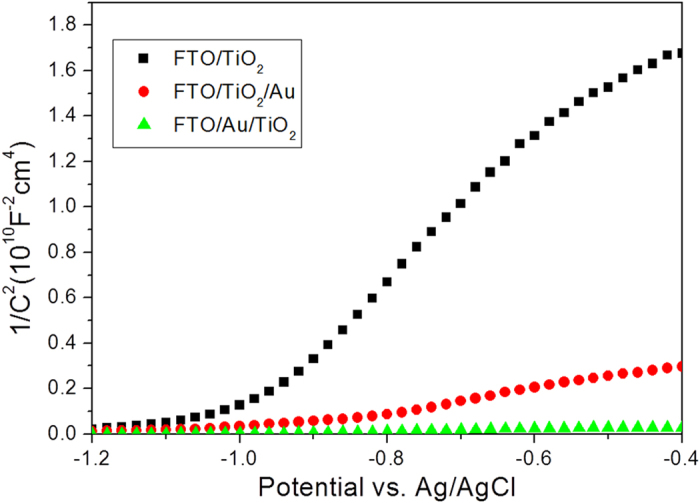
The MS curves of FTO/TiO_2_, FTO/TiO_2_/Au and FTO/Au/TiO_2_ photoelectrodes.

In order to investigate the underlying mechanism of this sequence-driven photocurrent direction behaviour under visible-light illumination, electrochemical impedance measurements were carried out. Capacitance was derived from the electrochemical impedance obtained at each potential with 5 kHz frequency in the dark. In addition to the Mott-Schottky (MS) plots of both hybrid photoelectrodes, one control sample of 10 nm of TiO_2_ is also depicted in Fig. 5. All these three samples exhibit positive slopes, indicating TiO_2_ component in the hybrid photoelectrodes still maintains n-type semiconductor. Compared to 10 nm of TiO_2_, both hybrid photoelectrodes show much smaller slopes in the MS plots, indicating much higher charge carrier densities^20^,^23^. The carrier density and flatband potentials can be quantified by the MS equation.
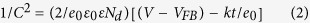
Where *e*_0_ is the electron charge, ε_0_ the permittivity of vacuum, ε the dielectric constant of TiO_2_ (75)^24^, *N*_d_ the dopant density, *V* the applied electrode potential, *V*_FB_ the flatband potential, and *k*t/*e*_0_ is a temperature-dependent correction term. The flatband potentials can be determined from the extrapolation of X intercepts in MS plots. The carrier density can be deduced from the slopes of MS curve using the equation.
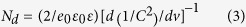


The width of the space-charge layer L_D_ at the photoelectrode/solution interface can also be derived from the MS plot relationship and be expressed as
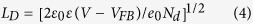


**Figure 6:**
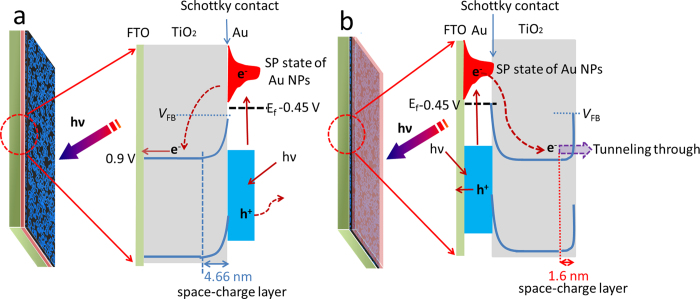
Schematic illustration of the visible-light excited charge-transferred process for Au/TiO_2_ bilayer structures: (**a**) FTO/TiO_2_/Au and (**b**) FTO/ Au/TiO_2_; here V_FB_ = Flat-band potential, E_f_ = Femi level.

Based on these three equations, the flatband potential, carrier density and the width of the space-charge layer can be calculated, as summarized in Table 1. It is clear that the flatband potentials for all these three samples are very close, around -1 V vs. Ag/AgCl. The carrier density is gradually increased in the TiO_2_ from 5.58 × 10^19^ cm^−3^ for bare FTO/TiO_2_ to 3.26 × 10^20^ cm^−3^ for FTO/TiO_2_/Au and then to 2.90 × 10^21^ cm^−3^ for FTO/Au/TiO_2_. The reason for TiO_2_ in FTO/Au/TiO_2_ has so much higher carrier density than that of TiO_2_ in FTO/TiO_2_/Au and FTO/TiO_2_ could be closely associated with the possible doping effect of Au during the heating process of FTO/Au/Ti films in air^25^,^26^,^27^. At the applied potential of −0.1 V vs. Ag/AgCl, the widths of the space-charge layer were calculated to be 11.57, 4.66, and 1.6 nm for FTO/TiO_2_, FTO/TiO_2_/Au and FTO/Au/TiO_2_, respectively. Based on these calculated data, the band diagrams of FTO/TiO_2_/Au and FTO/Au/TiO_2_ are drawn in Fig. 6. Because TiO_2_ is UV-active semiconductor, visible-light irradiation can only excite Au NPs. The Femi level of 5 nm of Au NPs is located around −0.45 V vs. reversible hydrogen electrode (RHE)^28^. Since the energy position of surface plasmon (SP) state is more negative than the conduction band of TiO_2_ (around 0 V vs. RHE, the difference between the Ag/AgCl and RHE in 1 M NaOH is 1 V), the photo generated electrons are easily transferred from the SP state of Au to the conduction band of TiO_2_ upon visible-light illumination. Due to the band bending formed at the interface between the photoelectrode and electrolyte, the photo generated electrons can only go through the outer circuit to the counter electrode for reduction reactions. Thus, the produced photocurrent is anodic. This visible-light excited charge-transfer process at the FTO/TiO_2_/Au photoelectrode is shown in Fig. 6a. For an FTO/Au/TiO_2_ photoelectrode, a similar visible-light excited charge-transfer process also happens. The only difference is that the photo generated electrons transferred at the conduction band of TiO_2_ are easily trapped within the energy well formed between the energy barriers of band bending and Schottky contact, as illustrated in Fig. 6b. These hot electrons should be very difficult to cross over the energy barriers of band bending or the Schottky contact, but it should be possible for them to tunnel through the TiO_2_ band bending to directly involve in the reduction reactions at the photoelectrode/electrolyte interface since the width of the space-charge layer is as thin as 1.6 nm for FTO/Au/TiO_2_ photoelectrode. The similar tunnelling effect has been reported on the protective TiO_2_ layer covered Si wafer^22^ and native SiO_2_ coated Si wafer^29^,^30^. If the TiO_2_ layer of FTO/Au/TiO_2_ photoelectrode increases to 100 nm, the thus-obtained photoelectrode still keeps cathodic photocurrent (Figure S7), suggesting the tunnelling effect still exists, not being dependence of the thickness of TiO_2_ layer.

Note that for FTO/Au/TiO_2_ photoelectrode, severe electron-hole recombination might be a key reason for the enhanced spike signals in Fig. 3d. In addition to the electrons tunnelling through the TiO_2_ band bending, some of the electrons will also flow back to FTO due to the direct contact between TiO_2_ and FTO on the interstitial space of 5 nm thickness of Au film. The subsequent recombination of these electrons with excited holes will result in the formation of the spike signal on the I-t curves (Fig. 3d). In addition, the trapped photo-generated electrons can remarkably increase the possibility of the electron-hole recombination, which can also contribute the spike transient current as demonstrated in Fig. 3d. Compared with the transient photocurrent in Fig. 3b,d, it is clear that the stationary photocurrent in Fig. 3d is much lower and the spike is much severer than those in Fig. 3b, implying the much heavier electron-hole recombination happened due to the flowing back and the trapped electrons under this condition. In contrast, the photo-generated electrons can quickly transfer from the conduction band of TiO_2_ to the conductive FTO substrate for FTO/TiO_2_/Au photoelectrode, therefore, the spike is almost disappeared in Fig. 3b. Not that the charging and discharging of the photogenerated surface states formed on TiO_2_ may also contribute to the spike in the I-t curves^31^, and detailed investigation on this issue is required in the following studies.

In summary, we have demonstrated an intriguing sequence-driven anodic to cathodic photocurrent phenomenon on a simply designed bilayer FTO/Au/TiO_2_ and FTO/TiO_2_/Au photoelectrodes. From the calculated results of MS plots, it is found that the carrier density of the TiO_2_ in the FTO/Au/TiO_2_ photoelectrode is more than 50 times larger than that of bare TiO_2_, which suppresses the width of space-charge layer to as thin as 1.6 nm. Thus, it provides a pathway for the trapped hot electrons tunnelling through the band bending for the reduction reaction. In contrast, the energy well is not existing for FTO/TiO_2_/Au photoelectrode, thus the generated hot electrons can directly flow to counter electrode for reduction reaction. As a result, the bilayer thin films with simply swapped sequence of Au and TiO_2_ can successfully realize the controlling of the photocurrent direction under visible-light irradiation. These findings and the proposed visible-light excited charge-transfer process may shed light on the potential applications of smart photo-electronic areas.

## Methods

### Chemicals

NaOH was purchased from Sigma-Aldrich and was used as received without further purification. The water was taken from a Millipore system.

### The fabrication procedure

A typical FTO/Au/TiO_2_ sample was fabricated as follow; a clean FTO was first put in an E-beam instrument vacuum chamber and pumped to 1 × 10^−6^ bar. Then the Au was sputtered on the conductive side of at FTO at 0.2 Å/s until the thickness reached 5 nm. The thickness of the sputtered film was monitored by the attached crystal balance. After that, the Ti was continually sputtered under the same vacuum conditions until the thickness of Ti reach 10 nm. Finally, the FTO/Au/Ti was transferred into a furnace and the temperature was raised from room temperature to 500 °C at a ramping rate of 2 °C/min, and then kept for 1 hour to make ensure the complete oxidation of Ti into TiO_2_. The FTO/TiO_2_/Au sample was fabricated following the reverse sequence as the FTO/Au/TiO_2_ described above.

### Characterization

The Temescal FC-2000 system was used for the sputtering of Au and Ti on the FTO substrate. UV-vis absorption spectra were recorded with a V650 spectrophotometer (JASCO). The X-ray diffraction (XRD) patterns were collected on a diffractometer (Miniflex, Rigaku). To avoid the interference of the FTO substrate, quartz plates was used for the sputtering Au and TiO_2_ for XRD analysis. X-ray photoelectron spectroscopy (XPS) was performed using an X-ray photoelectron spectrometer (a monochromatic Al KR X-ray source, Thermo Escalab 250). Scanning electron microscopy (SEM) and Energy-dispersive X-ray spectroscopy (EDS) mapping were performed on JEOL JSM-7001 F and JEOL JSM-6610, respectively. For better conductivity, several nanometers of Ir were sputtered onto the FTO/Au/TiO_2_ photoelectrode for SEM characterization.

### Photoelectrochemical measurements

Photoelectrochemical measurements were performed in a home-made one-compartment reactor with a quartz window, through which light was illuminated on the working electrode (Schematically illustrated in Figure S8). A three-electrode configuration was used with Pt plate, Ag/AgCl electrode, and FTO/Au/TiO_2_ or FTO/TiO_2_/Au as the counter, reference, and working electrodes, respectively. 1 M of NaOH solution (pH = 13.6) was used as the electrolyte. I-t curves were measured at −0.1 V vs. Ag/AgCl on an Electrochemical Workstation (CHI660d). In our experiment, the photocurrent was measured under irradiation from a 150-W Xe lamp (Newport) equipped with an air mass filter (Newport, AM 1.5 Global), calibrated with a Si diode to simulate AM 1.5 illumination (100 mWcm^−2^ = 1 sun). The illumination area was set by an aperture to 0.785 cm^2^. The Mott–Schottky analysis was performed in a three-electrode configuration in 1 M NaOH solution with 5 kHz frequency in the dark.

In the photoelectrochemical measurement, two half-reactions occur on the working and counter electrodes, respectively. During this process, electrons can be extracted or injected from the electrodes into the electrolyte solution. As shown in Figure S8, the electrolyte solution plays as a donor or acceptor to contribute or receive the electrons from the electrodes. Meanwhile, the electrolyte solution can also provide an ionic pathway to realize the closed circuit. Therefore, the chemical solution is necessary for the measurement of photocurrent.

## Additional Information

**How to cite this article**: Chen, H. *et al.* Switched photocurrent direction in Au/TiO_2_ bilayer thin films. *Sci. Rep.* **5**, 10852; doi: 10.1038/srep10852 (2015).

## Supplementary Material

Supplementary Information

## Figures and Tables

**Table 1:** The MS results of three types of photoelectrodes.

**Samples**	**E_fb_ (V vs. Ag/AgCl)**	**N_d_ (cm^−3^)**	**L_D_ (nm) at −0.1 V**
FTO /TiO_2_	−1	5.58 × 10^19^	11.57
FTO/TiO_2_/Au	−0.96	3.26 × 10^20^	4.66
FTO/Au/TiO_2_	−0.99	2.90 × 10^21^	1.60
