# Supplementary material for: Switched photocurrent direction in Au/TiO2 bilayer thin films
Source: Sci Rep. 2015 Jun 1;5:10852. doi: 10.1038/srep10852 (PMC4450582; doi:10.1038/srep10852)
Supplement: Supplementary Information [file srep10852-s1.doc]

# Supporting information

**Switched photocurrent direction in Au/TiO_2_ bilayer thin films**

Hongjun Chen^1^, Gang Liu^2^ & Lianzhou Wang^1^

^1^ Nanomaterials Centre, School of Chemical Engineering and Australian Institute for Bioengineering and Nanotechnology, The University of Queensland, QLD 4072, Australia

E-mail: [l.wang@uq.edu.au](mailto:l.wang@uq.edu.au)

^3^ Shenyang National Laboratory for Materials Science, Institute of Metal Research, Chinese Academy of Sciences, 72 Wenhua Road, Shenyang 110016, China

Email: [gangliu@imr.ac.cn](mailto:gangliu@imr.ac.cn)

To investigate the interaction between the Au NPs and TiO_2_, UV-vis was used to characterize these two photoelectrodes. The 5 nm of Au NPs and 10 nm of TiO_2_ by themselves are also fabricated on quartz plates as control samples for UV-vis characterization. As shown in Fig. S2, quartz/Au has a broad SPR peak from around 500 to 900 nm. This is because the local SPR interactions between the neighbour Au NPs which cause the SPR peak to be greatly red-shifted^1^. For quartz/TiO_2_, the typical absorption peak is located around 300 nm, which is ascribed to the photon excitation from valence band to conduction band^2,3^. Because both photoelectrodes have the same components, their UV-vis spectra are very similar, they not only have the typical absorption of TiO_2_ but also have the SPR peak of Au. Due to the interaction between the Au NPs and TiO_2_, both photoelectrodes exhibit obvious absorption from visible to near-infrared regime, which is much better than that of bare quartz/TiO_2_.

XRD was used to characterize the crystallinity of the photoelectrodes. As shown in Fig. S3, a strong broad diffraction peak around 21.5^o^ degree belongs to the underlying quartz substrate. One small diffraction peak located around 38.5 and another very weak diffraction peak around 44.8 ^o^ can be ascribed to <111> and <222> pattern of Au. The much higher intensity of Au <111> in quartz/Au/TiO_2_ than quartz/TiO_2_/Au should be directly connected with that the underlying quartz substrate can provide more suitable environment for Au deposition under certain growth direction than that of TiO_2_. However, the diffraction pattern of TiO_2_ is unfortunately not detected, which is probably due to the fact that TiO_2_ film is too thin or the formed TiO_2_ film is amorphous phase.

The morphologies of these two photoelectrodes were also characterized by SEM observations. The SEM image of FTO/TiO_2_/Au is shown in Fig. S4a, it can be clearly seen that many irregular patches with size around 10 to 50 nm are uniformly distributed on the surface. Due to low conductivity, several nm of Ir was sputtered on the surface of FTO/Au/TiO_2_. As shown in Fig. S4b, lots of small particles are uniformly distributed on the surface. Note that Due to the rough surface of FTO substrate and ultrathin layer of the Au and TiO_2_ (5 and 10 nm respectively), it is very difficult to elucidate the exact structure and to distinguish the shape and size of Au and TiO_2_ under our SEM operation conditions. In order to characterize the distribution of Au and TiO_2_, elemental mapping was also used for both photoelectrodes and found that the elements of O, Ti and Au were uniformly distributed on both photoelectrodes (data not shown).

Regarding the potential use of the simple bilayer structure design, we anticipate the fields of photo-induced transistors and photodectors. There were some previous studies in relevant fields. For instance, Park et al. studied individual Si nanowire based field effect transistors and reported a phenomenon of switched photocurrent direction (Nano Lett., 2005, 5, 1367).^4^ They found a positive current was generated if a Si nanowire was illuminated near the drain Ni electrode, whereas a negative current was produced when the irradiation moved to the source Ni electrode. The Schottky contact of Ni–Si junction resulted in the photocurrent and the polarity of the photocurrent was ascribed to the photoexcited electrons preferred to inject into the closest metal instead of crossing the Schottky barriers. In other examples, Au plasmonic nanostructures have been reported either to drastically improve the efficiency of graphene-based photodetectors (Nature Commun. 2011, 2, 458)^5^ or to realize multicolour photodection (Nature Commun. 2011, 2, 579).^6^ Based on these studies, we are expecting to make use of our bilayer structure in the relevant photo-induced devices.

For our bilayer electrode design, if considering the UV light and visible light as two inputs and two types of bilayer structures (FTO/Au/TiO_2_ and FTO/TiO_2_/Au) as other two inputs, the outputs are anodic or cathodic photocurrent. Based on different combination of inputs, the optical logic gating devices should be designed, which has similarity as the reported light-driven chemical logic gate of [Fe(CN)_6_]^4-^ complex modified TiO_2_ photoelectrode (J. Am. Chem. Soc. 2006, 128, 4550).^7^


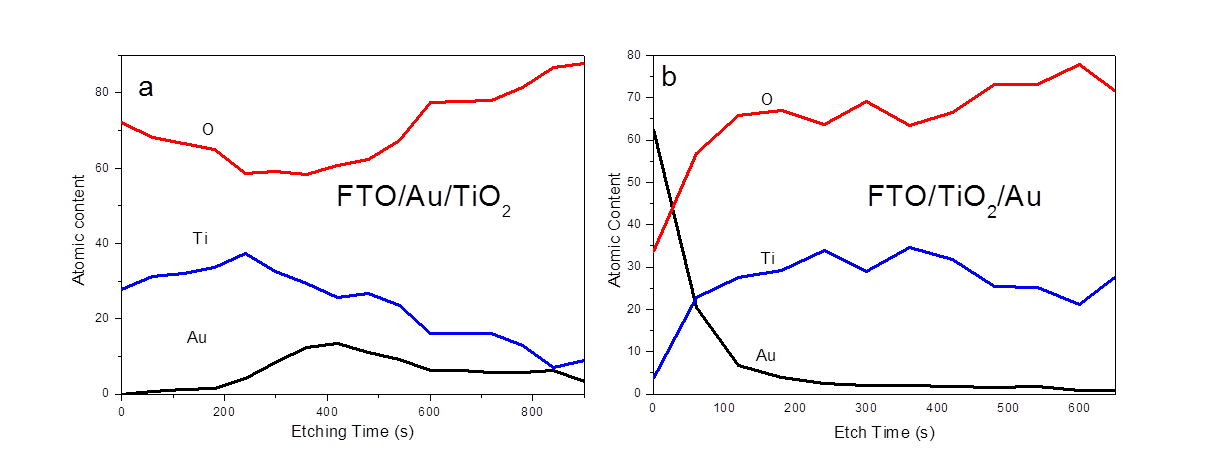


**Figure S1.** XPS depth profile of the Au/TiO_2_ bilayer structures. (a) FTO/Au/TiO_2_ and (b) FTO/TiO_2_/Au photoelectrodes.

**
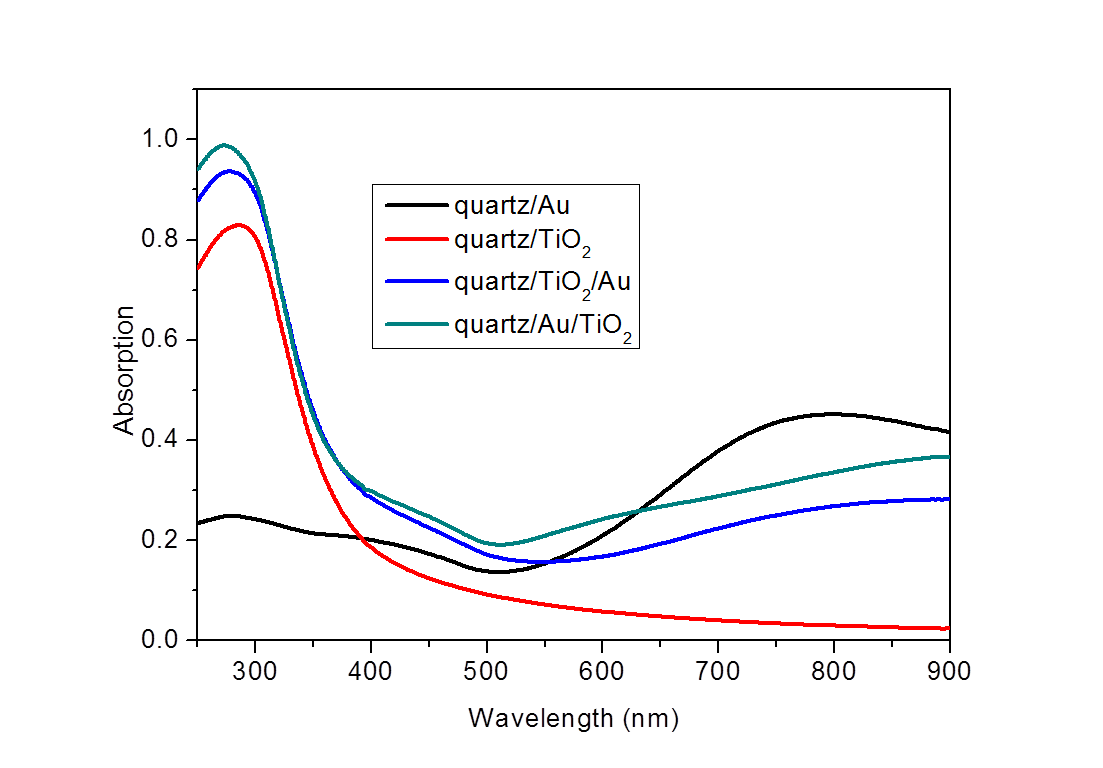
**

**Figure S2.** UV-vis spectra of the TiO_2_/Au and Au/TiO_2_ bilayer structures deposited on quartz substrates. For comparison, the spectra of quartz/Au, quartz/TiO_2_ are also shown herein.


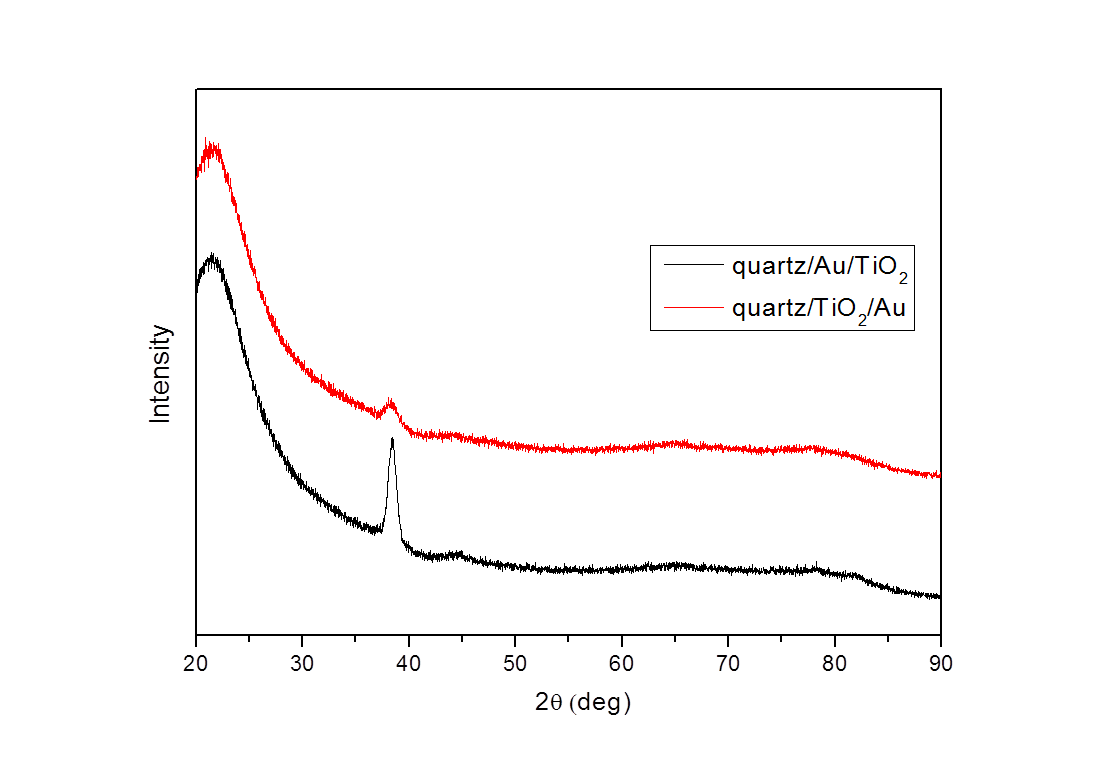


**Figure S3.** XRD patterns of the Au/TiO_2_ bilayer structures: quartz/Au/TiO_2_ (black line) and quartz/TiO_2_/Au photoelectrodes (red line).


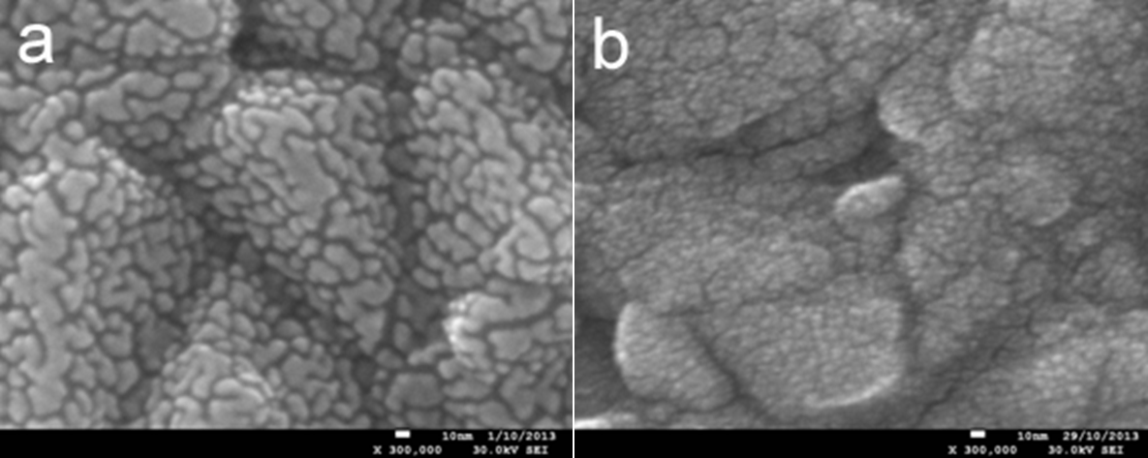


**Figure S4.** SEM images of the Au/TiO_2_ bilayer structures: (a) FTO/TiO_2_/Au and (b) FTO/Au/TiO_2_ photoelectrodes (top view).


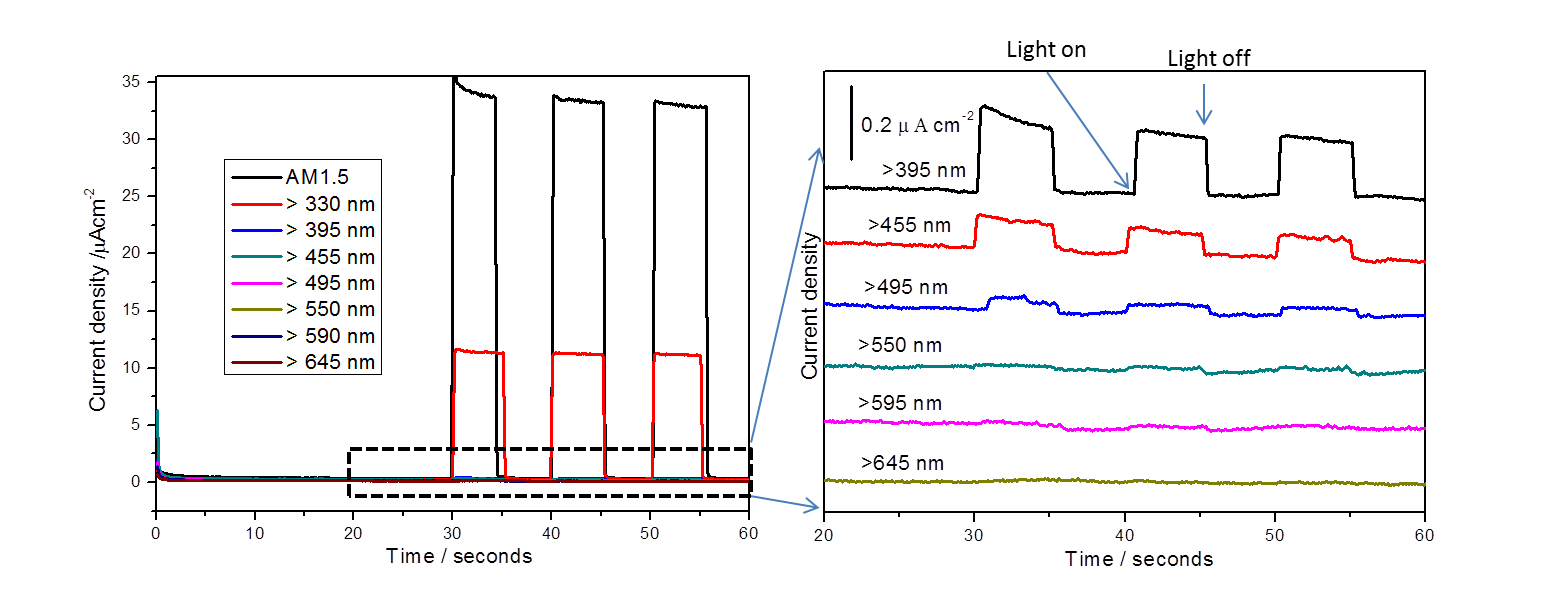


**Figure S5.** I-T curves of FTO/TiO_2_ photoelectrode under chopped light with different filter wavelengths. The right figure is the enlarged IT curves for the dashed rectangular part in left figure. Applied potential: -0.1 V vs. Ag/AgCl.


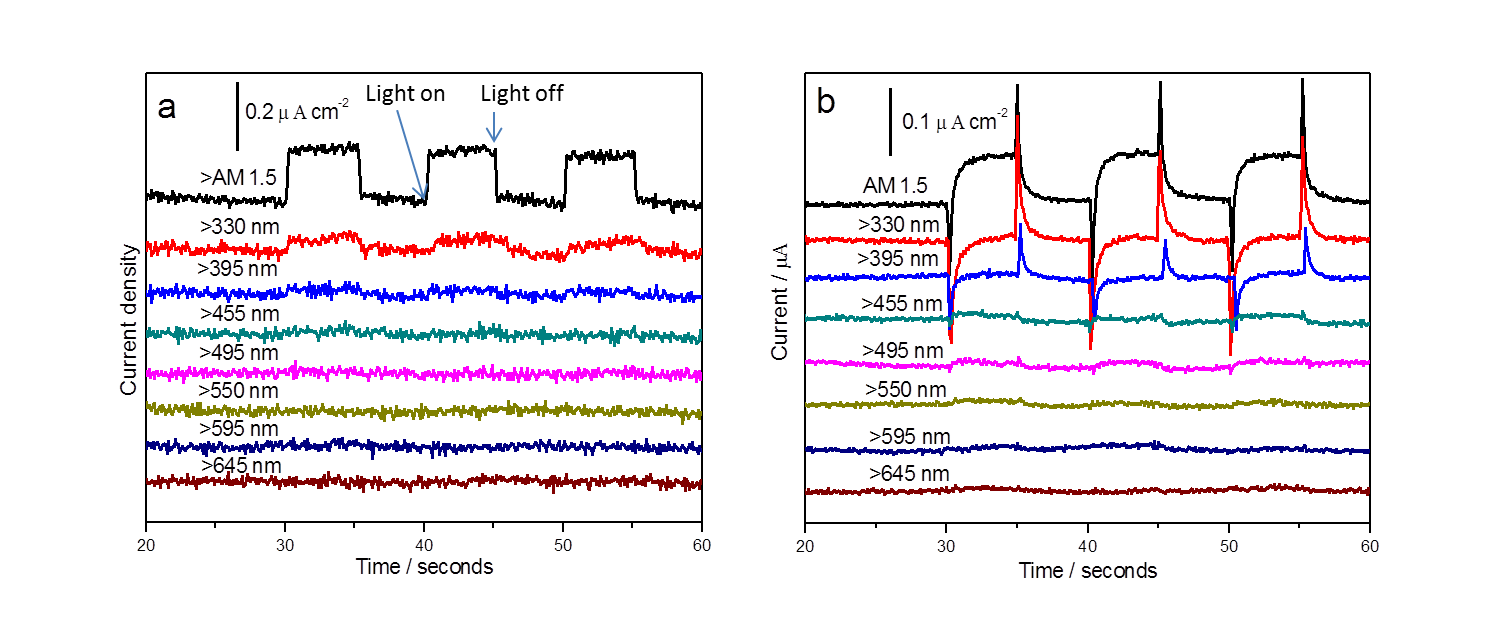


**Figure S6.** I-T curves of (a) FTO/Au (b) and bare FTO as photoelectrodes under chopped light with different filter wavelengths. Applied potential: -0.1 V vs. Ag/AgCl.

**Figure S7.** I-T curve of FTO/Au/TiO_2_ photoelectrode with 100 nm thickness of TiO_2_ under chopped light with filter wavelength of > 550 nm. Applied potential: -0.1 V vs. Ag/AgCl.


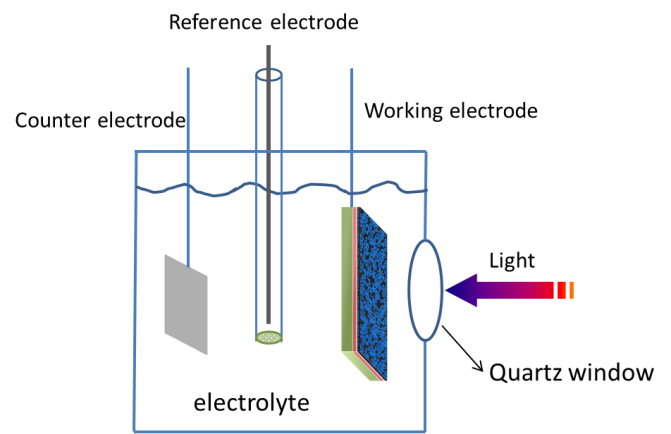


**Figure S8.** The sketch diagram of the photoelectrochemical measurement setup.

**References**

1. Chen, H., Wang, Y., Jiang, H., Liu, B. & Dong, S. Spontaneous formation of two-dimensional gold networks at the air-water interface and their application in Surface-Enhanced Raman Scattering (SERS). *Cryst. Growth Des*. **7**, 1771–1776 (2007).
2. Pan, J., Liu, G., Lu, G.Q. & Cheng H. M. On the true photoreactivity order of {001}, {010}, and {101} facets of anatase TiO_2_ crystals. *Angew. Chem Int. Ed.* **50,** 2133–2137 (2011).
3. Liu, G. *et al.* Nitrogen-doped titania nanosheets towards visible light response. *Chem. Commun.* 1383-1385 (2009).
4. Ahn, Y., Dunning, J. & Park, J. Scanning photocurrent imaging and electronic band studies in silicon nanowire field effect transistors. *Nano Lett.* **5**, 1367–1370 (2005).
5. Echtermeyer T.J. *et al.* Strong plasmonic enhancement of photovoltage in graphene. *Nature Commun.* **2**, 458 (2011).
6. Liu, Y. *et al.* Plasmon resonance enhanced multicolour photodetection by graphene. *Nature Commun.* **2**, 579 (2011).
7. Szaciłowski, K., Macyk, W. & Stochel, G. Light-driven OR and XOR programmable chemical logic gates. *J. Am. Chem. Soc.* **128,** 4550–4551 (2006).
